# Hypercalcemia in a Patient with CKD Undergoing Left Total Hip Arthroplasty

**DOI:** 10.34067/KID.0000000794

**Published:** 2025-09-25

**Authors:** Saud Alsaleh, Trevor Stevens, William Fissell

**Affiliations:** Vanderbilt University Medical Center, Nashville, Tennessee

**Keywords:** AKI, calcium, CKD, kidney failure

## Abstract

**Figure:**
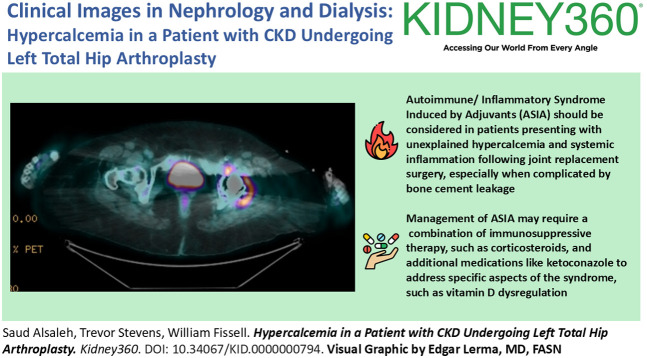

## Case Description

**Figure 1:**
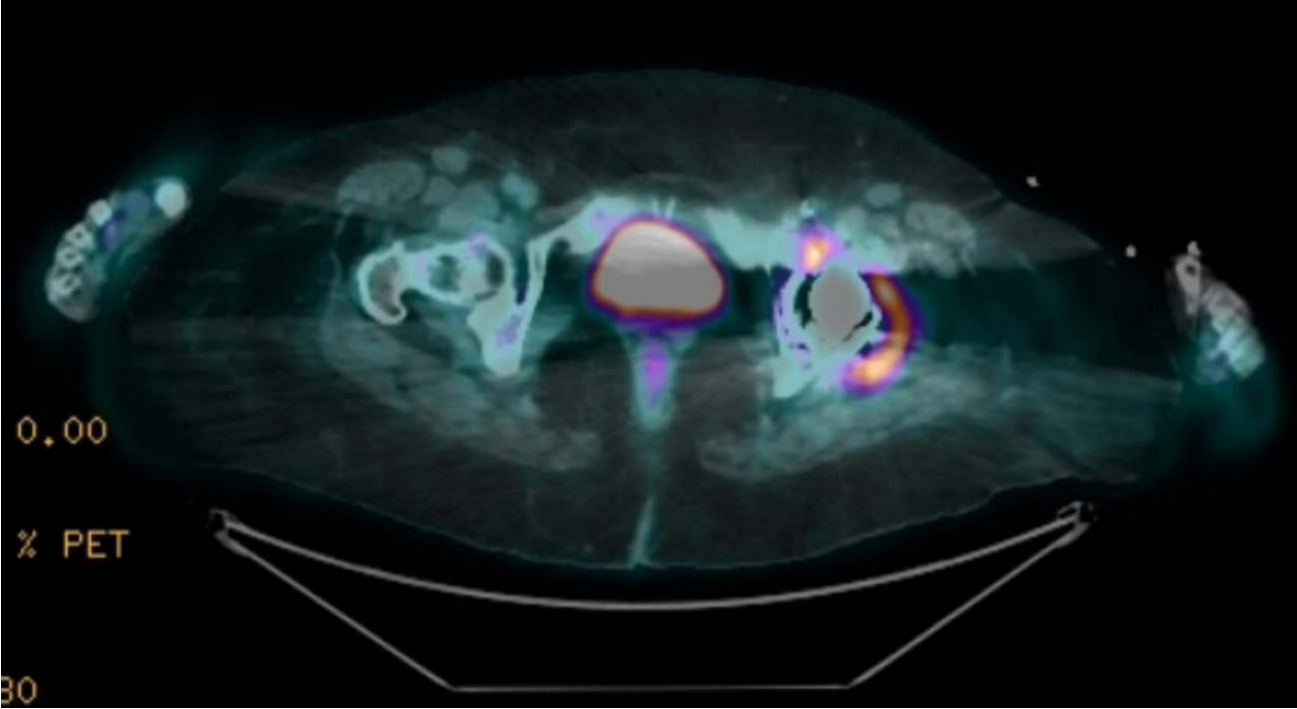
Positron emission tomography scan showing high radiotracer uptake in the left hip, suggesting increased metabolic activity.

A 69-year-old woman with CKD developed severe hypercalcemia and worsening kidney function after a left total hip arthroplasty complicated by bone cement leakage. She presented with confusion, elevated 1,25-dihydroxy vitamin D, parathyroid hormone–related peptide, and angiotensin-converting enzyme levels. Positron emission tomography scan revealed increased uptake around the left hip arthroplasty, left iliac adenopathy, and a nodular focus in the right rectus abdominis muscle (Figure 1). A diagnosis of autoimmune/inflammatory syndrome induced by adjuvants (ASIA) with a sarcoid-like reaction was considered. The patient was initially treated with prednisone 10 mg for 8 weeks, which led to improvement in calcium levels and kidney function, with creatinine improving from 2.2 to 1 mg/dl. However, with tapering the prednisone to 2.5 mg, calcium levels intermittently rose to 11.5–12 mg/dl.

During the corticosteroid taper, the patient developed chorioretinitis, requiring intravitreal corticosteroid injections. Ketoconazole was initiated to suppress vitamin D activation, but was not tolerated. Given persistent chorioretinitis, methotrexate 15 mg weekly was trialed; however, it remained refractory to treatment, and adalilumab add-on therapy is being considered.

## Discussion

This case highlights the potential complications of arthroplasty involving bone cement leakage, particularly in patients with preexisting CKD. The patient's presentation of persistent hypercalcemia, elevated inflammatory markers, and imaging findings suggestive of granulomatous inflammation point toward ASIA as a possible etiology. Polymethyl methacrylate, a common component of bone cement, may act as an adjuvant, triggering immune responses in genetically predisposed individuals.

The patient's initial response to prednisone, with improvement in calcium levels and kidney function, supports the diagnosis of ASIA. However, the recurrence of hypercalcemia and development of chorioretinitis on tapering of steroids emphasizes the chronic nature of this condition and the need for careful management. The addition of ketoconazole to suppress vitamin D activation, as well as immunosuppressant medications, demonstrates the complexity of treating ASIA and the potential need for multiple therapeutic approaches.

## Teaching Points

- ASIA should be considered in patients presenting with unexplained hypercalcemia and systemic inflammation following joint replacement surgery, especially when complicated by bone cement leakage.
- Management of ASIA may require a combination of immunosuppressive therapy, such as corticosteroids, and additional medications such as ketoconazole to address specific aspects of the syndrome, such as vitamin D dysregulation. Persistent hypercalcemia and inflammatory manifestations such as chorioretinitis may necessitate long-term immunosuppressive therapy, including methotrexate and biologic agents, to achieve disease control.

